# Animal Welfare and Food Safety Aspects of Confining Broiler Chickens to Cages

**DOI:** 10.3390/ani3020386

**Published:** 2013-05-13

**Authors:** Sara Shields, Michael Greger

**Affiliations:** 1 Humane Society International, Farm Animals, 2100 L St. NW, Washington, DC 20037, USA; 2 Humane Society of the United States, Farm Animals, 2100 L St. NW, Washington, DC 20037, USA; E-Mail: mgreger@hsus.org

**Keywords:** broiler, welfare, cage, food safety, behavior, stocking density, leg problems

## Abstract

**Simple Summary:**

In commercial chicken meat production, broiler chickens are usually kept on the floor in ware-house like buildings, but the use of cages is becoming more common. Confining chickens to cages is a welfare problem, as has been thoroughly demonstrated for laying hens used for egg production. Caged broiler chickens may suffer from poor bone strength due to lack of exercise, feather loss, and restriction of natural behavior. There are also potential food safety concerns associated with the use of cages. While cages may provide an economic advantage in some geographical regions of the world, the severe, inherent disadvantages should also be considered before cages are more widely adopted in the global broiler chicken industry.

**Abstract:**

In most areas of the world, broiler chickens are raised in floor systems, but cage confinement is becoming more common. The welfare of broiler chickens in cages is affected by movement restriction, poor bone strength due to lack of exercise, and prevention of key behavioral patterns such as dustbathing and ground scratching. Cages for broiler chickens also have a long history of causing skin and leg conditions that could further compromise welfare, but a lack of controlled studies makes it difficult to draw conclusions about newer cage designs. Cage environments are usually stocked at a higher density than open floor systems, and the limited studies available suggest that caging may lead to increased levels of fear and stress in the birds. Further, birds reared on the floor appear less likely to harbor and shed *Salmonella*, as litter may serve as a seeding agent for competitive exclusion by other microorganisms. Cages for laying hens used in egg production have met with substantial opposition due to welfare concerns and caging broiler chickens will likely be subject to the same kinds of social disapproval.

## 1. Introduction

Litter-bedded floor systems are common for raising broiler chickens used for meat production. In contrast, the egg industry has relied heavily on battery cages—small, wire enclosures that typically hold five to ten laying hens. Although cages for broiler chicken production have been available for many years, they were not widely adopted because heavy broiler chickens are prone to leg deformities [1,2,3], breast blisters [4], and other skin imperfections such as enlarged feather follicles [5] due to abrasion against the wire cage floor [6,7,8] and these problems have adversely affected meat quality [7,9]. Another problem is the comparatively short time period that broiler chickens are confined to cages before they reach market weight, and the concomitant labor requirements associated with moving chickens into and out of cages [4].

**Figure 1:**
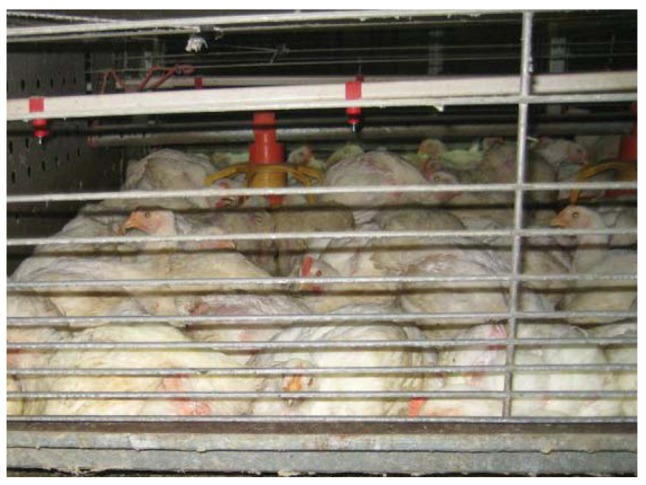
Caged broiler chickens (Photo by Sonia Faruqi).

Despite the obstacles, interest in developing a cage system that works well for broiler chickens has been ongoing since the 1960s [10]. A variety of cage floor materials have been tested including plastic tubing; plastic and metal mats [5,11]; rubber-covered nylon [12]; bamboo [8]; wire, steel and plastic mesh; perforated Styrofoam; padded doweling [13]; polyester urethane foam [14]; and wooden slats [15]. In the early 1970s a composite mesh floor material was patented, which helped solve earlier problems with breast blisters and skin imperfections [6]. Large colony cages (Figure 1) now have plastic-covered nylon floors that are less abrasive, and manufacturers claim they prevent breast blisters, folliculitis, and wing and leg problems. Collection of broiler chickens at the end of the growing period can now be automated; cage floors open to drop birds onto a conveyer belt below that moves them to the end of the house where they are crated for transport.

In certain geographical regions, economic factors favor the production of broiler chickens in cages. In countries where litter must be imported (and is therefore expensive) cages reduce costs associated with purchasing, removal, and disposal of litter. Because cages are stacked vertically, they permit a larger number of animals to be raised together in one building. In tropical counties, heat enters the house through the roof, and having a greater number of animals in the building lowers the per bird cost of overhead insulation [6]. In the United States, broiler chickens are grown until they reach approximately 2.5 kg, but in hot climate countries such as the Gulf region and Saudi Arabia, broilers are grown to a market weight of just 1.5 kg. Breast blisters are not as problematic in chickens grown to a lighter weight [2]. Broiler chickens in Russia have been bred with thicker shanks and longer toes, providing more support for the caged bird’s body and a lower incidence of breast blisters [7].

Since 2008, markets for broiler chicken caging systems have been growing in Russia, the Middle East, and several Asian countries, including China [16,17]. Cages are also used in Africa [18], India [19,20], and Eastern European countries [3]. However, it appears that not all broiler chickens are being raised in the newer colony cage designs. For example, a study of 46 poultry farms in Ondo State, Nigeria found that a quarter of broiler farmers reared their chickens in simple battery cage systems [18].

There have been few scientific studies of the effects of cages on the welfare of broiler chickens, but those that have been conducted suggest that, similar to cages for egg-laying hens, cages for broiler chickens present a number of welfare concerns.

Given the welfare disadvantages of cage production and the ensuing social disapproval, barren battery cages have been legislated against in several US states [21,22] and throughout the entire European Union [23]. Animal protection groups have launched major campaigns against the use of cages in all the developing economies of the world. The welfare of caged broiler chickens is justifiably likely to attract as much concern as cages for laying-hens have in the egg industry. 

## 2. Spatial Restriction

The obvious effect of the limited space in cages is restriction of movement. Although walking [24], movement [25] and total space use [26] decline with age, broiler chickens are relatively active throughout the day in floor pens. While distance moved can be highly variable between individuals, Lewis and Hurnik (1990) found that at five weeks of age birds traveled an average of 8.8 m every hour and 212 m daily in pens measuring 1.2 by 1.7 m [27]. In another study, floor-raised chickens in commercial poultry housing regularly moved several meters from one lying spot to another [28]. 

Total area covered and total distance travelled are two different aspects of chicken mobility that could be affected by enclosure size. Leone *et al.* (2010) and Leone and Estevez (2008) found that total area covered increased with enclosure size from 1.5 to 4.5 m^2^, regardless of stocking density or group size [25,29]. Although they did not find that broilers in progressively larger *square* [25] enclosures traveled a greater distance, they did find that chickens in progressively *longer* [29] enclosures travelled greater total distances as they were provided more space, as long as group size was held constant. This could mean that the effect of space allowance on total distance travelled depends on the configuration of the available space and the level of crowding, but since few studies have tracked distance travelled, more research is needed to further understand the relationship between enclosure size and movement restriction.

Caging broiler chickens also alters the types of behavior that broiler chickens display compared to floor systems. Fouad *et al.* (2008) conducted a large trial with two commercial flocks each of about 12,375 broiler chicks raised to six weeks of age in either cages or on the floor. They found that floor-raised broiler chickens were more often seen walking, lying, and pecking compared to those in cages. In contrast, caged broilers stood and drank significantly more often, a finding explained by the replacement of walking activity with standing behavior [30]. The lack of free space appears to constrain activities that broiler chickens would otherwise choose. 

Restriction of movement in cages can have tangible consequences on the physical integrity of animals. Laboratory tests have demonstrated that the bones of broiler chickens reared in cages have significantly lower breaking strength. Several studies have found that the humeri are stronger in broiler chickens raised on the floor as compared to those raised in cages [11,12,31,32]. Tolon and Yalcin (1997) found that birds reared in cages with plastic mesh floors had shorter tibia and humeri as compared to birds raised in floor pens, and humerus weight was greater for birds reared on the floor [33]. The authors of these studies attributed wing bone differences to difficulty expressing normal wing-related activity such as flapping behavior in the cage environment [11,33]. Indeed, reducing cage height has been shown to reduce humerus breaking strength [34].

## 3. Stocking Density

The stocking density in floor systems is known to influence the behavior of broiler chickens. Buijs, Keeling, Vangestel, Baert, and Tuyttens (2011) observed broiler chickens stocked at eight different stocking densities between 8 and 72 birds per 3.3m^2^, and found more bouts of sitting behavior at the higher stocking densities. They also found more disruption of lying and sitting bouts during the last week of rearing and that more birds made adjustments of their sitting and lying posture at the higher stocking densities [35]. Bokkers, de Boer, and Koene (2011) measured the amount of floor space occupied by a six-week-old broiler chicken during the expression of a variety of different behavior patterns and found that keeping birds at a relatively high stocking density resulted in compression of the body surface, due to pressure on the feather and soft tissue cover. As determined using overhead photographs taken of broilers enclosed with either eight (1,250 cm^2^/bird) or 16 birds (625 cm^2^/bird) per pen, birds were found to occupy less space at the higher stocking density. The mean body space for sitting idle was 636 cm^2^ in the low density pen, and 514 cm^2^ in the high density pen, while standing while stretching, which required the largest space allowance, was 763 cm^2^ in the low density pen and 707 cm^2^ in the high density pen. The authors concluded that stocking densities in excess of 39.4 kg/m^2^ would suppress behavioral expression [36]. A 2007 review concluded that when broiler chickens are raised on the floor with less than approximately 34 to 38 kg/m^2^, the negative consequences include lower final body weight, less feed intake, poor feathering, and more scratches and bruising [37].

Research on stocking density allowances for broiler chickens have found greater mortality for part of the rearing period, a higher incidence of leg problems, more contact dermatitis, increased carcass bruising, disrupted resting behaviour, and decreased locomotion and ground pecking in floor systems stocked at 40 kg/m^2^ as compared to 34 kg/m^2^ [38]. The maximum permitted stocking density in the European Union is 33 kg/m^2^, with derogations permitting up to 42 kg/m^2^ if specific air quality, temperature and humidity requirements are met [39]. In floor systems the body of the chicken is in contact with on the litter/manure surface when the bird rests. In cages however, the air space underneath the floor is thought to prevent the buildup of heat and ammonia [17]. Thus, the recommended stocking density for caged broiler chickens can be very high, with some poultry equipment manufacturers suggesting 50 kg/m^2^. For a chicken growing to 1.5 kg, this is 33.3 birds/m^2^ or 300 cm^2^ of space per bird. By contrast, industry guidelines in the United States stipulate that caged laying hens should receive 432 cm^2^ (67 in^2^) of space per bird [40], and the legal minimum for laying hens in the European Union is 750 cm^2^ [23]. 

The value of additional space to the birds themselves has been determined using motivational assessments. Buijs, Keeling, and Tuyttens (2011) calibrated the height of barriers that broiler chickens would cross to access feed when they were and were not food deprived to determine a “low” barrier height and a ‘high’ barrier height. They then used these barriers to determine how hard broiler chickens were willing to work in order to access to more pen space. Spatial preference was determined by monitoring bird movements from one enclosure with 14.7 birds/m^2^ to another enclosure of 9.3, 12.1 or 14.7 birds/m^2^. The experiments showed that the lower the stocking density on the other side of the pen, the more birds crossed over the barrier to move to that side. Broiler chickens preferred the lower stocking density even when they had to cross over a barrier that was high enough to deter 20–25% of birds from crossing to access feed after six hours of food deprivation. The researchers concluded that broiler chickens prefer more space than the 42 kg/m^2^ provided in their study, and that a lower stocking density is important to broiler chickens [41].

## 4. Leg Disorders

Rapid growth of muscle on an immature skeleton leaves broiler chickens prone to bone, joint, and ligament disorders. As a result, broiler chickens often suffer from leg deformities and lameness [42,43,44], and leg problems severe enough to hinder walking ability are known to be painful [45,46].

**Figure 2:**
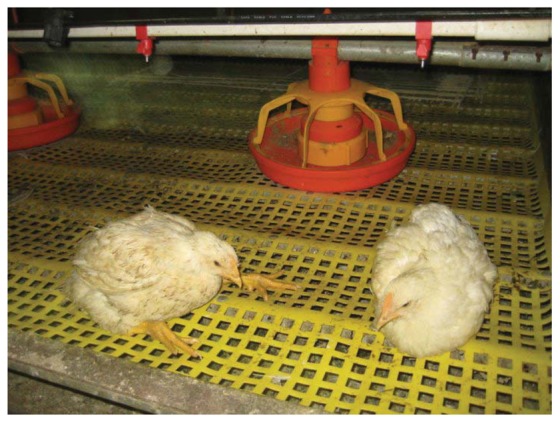
Broiler chickens in cage with plastic flooring (Photo by Sonia Faruqi).

Lack of activity has been associated with gait and skeletal disorders [1,47]. Broiler chickens housed in cages have a greater prevalence of gait problems, impaired walking ability and leg abnormalities (Figure 2) than those raised on the floor [1,30,48]. Starting broiler chicks in battery cages has been associated with a higher incidence of twisted legs before eight weeks of age [49]. Rizk *et al.* (1980) found that broilers reared in battery cages had a 20 to 60% incidence of perosis-curled toes compared to 12 to 13% for birds in floor-pens [48]. Reece *et al.* (1971) used a plastic mat to cover the wire-mesh bottom of cages, but still found significantly more of a perosis-like condition in the joint between the tibia and the metatarsus [4]. Haye and Simons (1978) tested the effect of various types of cage floor materials on leg problems, including plastic sheets, mats on wire, and covered wire mesh, and found that broiler chickens had more twisted legs in cages as compared to floor reared birds on litter, although metal wire and perforated metal sheets were worse than plastic or plastic-coated wire [1]. Using gait scoring from 0 (no gait impairment) to 5 (total immobility), Fouad *et al.* (2008) found that the proportion of birds with score 0 was higher in floor reared birds (72%) compared to those confined to a cage (48%), however no details on the floor or cage type tested were reported [30]. Cage equipment manufacturers claim that their designs no longer cause leg problems, and so additional published research is needed. Given the association between lack of exercise and leg problems, if cages restrict movement then leg condition may be problematic regardless of floor type.

## 5. Lack of Loose Litter

One of the advantages of floor systems is that chickens can express scratching and dustbathing behavior in the loose substrate, often wood shavings. Dustbathing keeps the birds’ plumage in healthy condition by balancing lipid levels in the feathers [50,51]. Studies of laying hens show that dustbathing is performed approximately once, every other day [52]. During dustbathing episodes, birds crouch, lie in, and rub dust through their feathers before standing and shaking off the loose particles. Light and heat trigger dustbathing, as does the presence of a friable, dusty substrate [50]. However, even when deprived of these normal eliciting stimuli, Junglefowl will still attempt to dustbathe on wire floors [53]. In behavioral experiments, Junglefowl deprived of dustbathing substrate will compensate by dustbathing more vigorously when eventually given access to a suitable substrate [54]. Dustbathing behavior is seen in young broiler chicks even in the first week of life [55]. Although there has been a report of dustbathing deprivation leading to stress in laying hens [56], Widowski and Duncan (2000) suggest that dustbathing is not driven by a need, but is rather a pleasurable activity [57]. Since good welfare is dependent on both an absence of suffering *and* the presence of enjoyable activity [58], the inability of caged broiler chickens to perform dustbathing behavior in loose litter may be a detriment to their welfare.

When roaming freely outdoors, chickens often engage in foraging and scratching behavior. Chickens scratch in a variety of substrate types, from dry earth to vegetated groundcover. Both Junglefowl [59] and domestic chickens [60] in a naturalistic environment will spend approximately 50% of their active time foraging. Chicks begin to express exploratory pecking shortly after hatching [61], and studies of feral fowl report that broods aged 3–7 weeks, like adult birds, spent about half their daily time budget in foraging behavior [60]. Chickens will continue to work for their food (using an operant conditioning panel in a Skinner box) even when identical feed pellets are freely available in a feeder [62], demonstrating that the appetitive component of foraging, not just the consummatory phase, is important. Indeed, despite being fed three times a day, captive bred Junglefowl continued to forage when released into a large wooded area of tall trees and low bushes, spending 60% of the active part of their day ground pecking and 34% ground scratching [59]. Broiler chickens reared in floor systems can perform normal ground scratching in litter, but caged birds are limited to scratching on the wire cage floor.

## 6. Growth

Research on productivity of broiler chickens in cages shows mixed results. Some studies show that floor reared broilers have significantly higher growth rates and heavier final body weights compared to cage reared groups [2,30,33,63] while others find no significant difference in weight gain [5,10,64,65]. At least one study reported significantly higher growth in cages as compared to floor systems [66]. Hypes, Carpenter, Peterson, and Jones (1994) found that when cage reared chickens were moved to the floor at 21 days of age, they displayed compensatory growth, but still weighed less than their counterparts who were reared on the floor for the entire 42 day period [67]. The disparity in results may be due in part to differences in the cage floor material used, as at least one study showed lighter body weights for birds raised on wire mesh compared to birds reared on litter, steel or plastic mesh, perforated floors (made of either wood, styrofoam or plastic) or doweling (rigid, rotating or padded) with 1.91 cm of open space between horizontal components [13]. Differences in growth rate also depend on stocking density, as studies show that crowding in both floor systems [4] and cages [68] can reduce growth rates.

Growth rate is also influenced by feeding behavior. In one study, floor-reared birds were recorded eating more often than caged broilers and showed significantly higher body weight gain [30], suggesting that feed consumption may increase in floor reared flocks. However, at least one other study reports no difference in feed consumption between broiler chickens reared on the floor and in cages [65].

## 7. Fear and Stress

Rearing in a cage environment has been shown to cause birds to react in a more fearful way when tested in experimental trials. Using tonic immobility, novel object, and novel environment tests, several studies have found that laying hens kept for egg production are more fearful when confined in cages compared to those kept in cage-free housing [69,70,71,72]. This robust result has been shown to hold true for broiler chickens kept in cages as well [30].

Only one study has taken measures of stress on broiler chickens in cages. Fouad *et al.* (2008) found greater heterophil to lymphocyte ratios in caged birds (six to a cage with 500 cm^2^ floor area/bird), compared to those of birds reared in floor pens [30]. Rodenburg *et al.* (2008) noted that laying hens can move away from barn staff and other birds in cage-free systems and distance themselves from potential threats, whereas in cages there is limited space for avoidance of people or cage-mates, and this may help explain why fear and stress differ between cage and floor reared birds [72].

## 8. Plumage Deterioration and Mortality

The feathers of birds insulate the body against heat loss and protect the underlying skin [73]. For laying hens, feather loss is generally worse in cages compared to other systems [74]. The same appears to hold true for broiler chickens kept in cages. Edens *et al.*, 1999 found better feathering in floor-reared birds compared to those raised in cages using the Broilermatic^®^ Cage System [10]. Feather loss or damage may be caused by abrasion with the cage wire [10] or from crowding, as has been demonstrated in experiments with laying hens [75,76]. 

Several recent studies have compared mortality rates of broiler chickens in different growing environments. A study in the United States using the Broilermatic^®^ Cage System [10], and studies in India [20], Egypt [30], and Nigeria [64] all found that mortality was not statistically different between broiler chickens reared in cages compared to those reared on the floor. 

## 9. Food Safety

In the egg industry, caging chickens has been definitively tied to increased food safety risk. Many factors have been cited to explain the association between cage housing and *Salmonella* in the egg industry, including larger flock sizes, higher stocking densities, more rodent and insect disease vectors, and greater difficulty cleaning and disinfecting the cage equipment itself [77]. Other potential mechanisms include impaired acquisition of normal gut flora, compromised gastrointestinal function, and physiological stressors.

Intuitively one might assume that hens reared in cage-free settings would harbor and spread fecal pathogens such as *Salmonella* more than birds suspended in cages and thereby separated from their waste. While cage housing can successfully break the lifecycle of intestinal parasites [78] such as helminthes [79] and may decrease *Campylobacter* flock persistence [80], ironically, access to fecal matter may actually decrease the threat of *Salmonella* by serving as a seeding agent for competitive exclusion microorganisms [81].

The capacity of used litter to shorten the carrier state of chickens and suppress *Salmonella* shedding and transmission was demonstrated more than 40 years ago [82,83]. This may explain why *Salmonella* infection rates can decline much more rapidly in chickens placed on bedding compared to those kept in cages [84]. Santos *et al.* (2008) found significantly lower intestinal *Salmonella* colonization in broilers reared on litter compared to those in cages [81]. They conclude: “Broilers raised on litter may have achieved lower cecal *Salmonella* populations than caged birds because access to litter may have modulated the intestinal microflora by increasing competitive exclusion microorganisms, which discouraged *Salmonella* colonization.” 

Though experimental data have been mixed [85,86], such enhanced resistance could explain why significantly lower levels of *Salmonella* were found in the excreta of cage-free birds in a study of 90 layer flocks in New Jersey, USA. All of the positive samples from cage-free birds contained relatively few *Salmonella*, with most probable counts <10 per g, whereas 50% of the composite samples from caged birds yielded values exceeding 10,000 per g. Likewise, *Salmonella* was found in old waste samples from caged birds, but not from cage-free. These findings are suggestive that cage housing may also promote *Salmonella* shedding in commercial settings [87].

Providing hens with access to bedding may also prevent *Salmonella* infection by improving gastrointestinal function. The gizzard pH of chickens raised on litter has been found to be significantly lower than that of caged birds. This may be because the coarse components of litter mechanically stimulate the proventriculus (glandular stomach) to produce more hydrochloric acid. The gizzards of caged chickens have also been found to be comparatively underdeveloped, attributed to the relative lack of insoluble fiber stimulation of the muscular organ [81]. A significantly higher incidence of ileal grain chips found in caged chickens is indicative of inferior gizzard function [85]. Because *Salmonellae* are not very acid tolerant [88], pecking at wood shavings may therefore play a major role in reducing cecal *Salmonella* colonization by improving the barrier function of both chambers of the stomach [81].

The only source of fiber for caged chickens is the nonstarch polysaccharides in their feed, which may not provide sufficient fiber to sustain normal intestinal development and function [81]. Greater insoluble fiber consumption may explain greater jejunum villus height, villus area, villus height-to-crypt depth ratio, and mucosal depth in litter-reared birds. This increased intestinal efficiency may explain the reduced ileal and jejunal mass in litter-reared broiler chickens, which may improve microbial stability and lower the pathogen load [81]. Enhanced peristaltic emptying alone may contribute to the control of *Salmonella* populations [89] by sweeping pathogens out of the bowel [90].

Faster declines in *Salmonella* shedding have been noted in experimentally infected cage-free hens compared to those confined in conventional cages even in barren environments, though, without access to litter [91]. This suggests additional factors, such as physiological stress, may be playing a part in the role of housing systems in *Salmonella* risk [92].

Increasing evidence indicates farm animal stress in general may have detrimental effects on the safety of the food supply [93]. Although in certain circumstances stress may enhance immune function, the bulk of evidence suggests that chronic or prolonged stress generally promotes the pathogenesis of infectious disease via a variety of potential mechanisms [94]. These include immunosuppression, via corticosterone [95] or resource allocation tradeoffs [96], stress-induced perturbations of intestinal microbiota or gut epithelium integrity [97], and the effects of neurohormones such as catecholamines on bacterial growth [98]. At the same time stress-related corticosteroids may be impairing a chicken’s immune response, the stress hormone norepinephrine released from adrenergic fibers lining the bird’s gut [99] may boost the growth rate of *Salmonella* by orders of magnitude [100] and increase colonization and systemic spread [101].

## 10. Conclusions

While the welfare of broiler chickens in floor systems is not without concern, the advantages of a cage-free system for broiler chicken production are more total space, greater opportunity for exercise and improved bone health compared to cage production. Birds kept in litter-based systems are able to express more of their natural behavior, including ground scratching and dustbathing. Broiler chicken cage equipment manufacturers claim that they have overcome past problems with leg and integument disorders, but this is difficult to verify without published evidence. Moving from a floor system to a cage system does not improve the overall welfare of birds raised for meat, and this important consideration should be carefully weighed in future decisions regarding expansion of cage systems for broiler production.
